# St. Bartholomew's Hospital

**Published:** 1903-12-19

**Authors:** 


### ST. BARTHOLOMEW'S HOSPITAL.

We have received the following communications:—

*To the Editor of* The Hospital.

Sir,—The House Committee and Committee of Treasurer and Almoners of this hospital have decided unanimously to recommend the Governors to take steps to ascertain the possibility of acquiring a further addition to their site. They have adopted this course in view of the great and general encouragement thereto given to them in the principal organs of the press and in other quarters. They believe that they have the unanimous support of their medical staff in the action they have decided to take in regard to this matter.

*To the Editor of* "The Hospital"

St. Bartholomew's Hospital, December 10th, 1903.

Sir,—We, the members of the medical and surgical staff of St. Bartholomew's Hospital, wish it to be known that we support most cordially the appeal of the governing body for funds.

We beg to state that, in our opinion, the first essential is the acquisition of more land, the next the erection of new buildings, and the ultimate reconstruction of the whole hospital on the extended area.

In pursuance of this scheme we are in hearty agreement with the Governors in their proposal to acquire more land and to erect first, and without further delay, the new out-patient block, on account of the urgent need of the departments which will be included in this building.

The acquisition of more land by the Governors will necessitate the reconsideration of all previous plans for wards and other new buildings, and will enable St. Bartholomew's to become one of the most complete hospitals of modern times.

Upon the response to this appeal will necessarily depend the future of the hospital.

We are, yours faithfully,

| | | |
|---|---|---|
| (Signed) | Samuel Gee. | John Langton. |
| | Dyce Duckworth. | Harrison Cripps. |
| | T. Lauder Brunton. | W. Bruce Clarke. |
| | Norman Moore. | Anthony Bowlby. |
| | Samuel West. | C. B. Lockwood. |
| | J. A. Ormerod. | D'Arcy Power. |
| | W. P. Herringham. | H. J. Waring. |
| | H. H. Tooth. | W. McAdam Eccles. |
| | A. E. Garrod. | R. C. Bailey. |
| | James Calvert. | W. H. H. Jessop. |
| | F. H. Champneys. | T. Holmes Spicer. |
| | W. S. A. Griffith. | A. E. Cumberbatch. |

